# Ultrasound-guided lesser occipital nerve combined with great auricular nerve block for vestibular schwannoma craniotomy via a suboccipital retrosigmoid approach: a prospective, double-blind randomized controlled trial

**DOI:** 10.1186/s12871-024-02642-2

**Published:** 2024-07-20

**Authors:** Tianzhu Liu, Jiuhong Liu, Liu Yang, Zongfang Wu, Yang Zhang, Feng Gao

**Affiliations:** Department of Anesthesiology and Pain Medicine, Hubei Key Laboratory of Geriatric Anesthesia and Perioperative Brain Health, and Wuhan Clinical Research Center for Geriatric Anesthesia, Tongji Hospital, Tongji Medical College, Huazhong University of Science and Technology, Wuhan, China

**Keywords:** Ultrasound-guided, Lesser occipital nerve block, Great auricular nerve block, Vestibular schwannoma resection, Suboccipital retrosigmoid craniotomy

## Abstract

**Purpose:**

This aim of this study was to investigate the analgesic efficacy and safety of lesser occipital nerve combined with great auricular nerve block (LOGAB) for craniotomy via a suboccipital retrosigmoid approach.

**Methods:**

Patients underwent vestibular schwannoma resection via a suboccipital retrosigmoid approach were randomly assigned to receive ultrasound-guided unilateral LOGAB with 5 ml of 0.5% ropivacaine (LOGAB group) or normal saline (NSB group). Numeric rating scale (NRS) scores at rest and motion were recorded within 48 h after surgery. Mean arterial pressure (MAP), heart rate (HR), opioid consumption and other variables were measured secondly.

**Results:**

Among 59 patients who were randomized, 30 patients received ropivacaine, and 29 patients received saline. NRS scores at rest (1.8 ± 0.5 vs. 3.2 ± 0.8, *P* = 0.002) and at motion (2.2 ± 0.7 vs. 3.2 ± 0.6, *P* = 0.013) of LOGAB group were lower than those of NSB group within 48 h after surgery. NRS scores of motion were comparable except for 6th and 12th hour (*P* < 0.05) in the LOGAB group. In LOGAB group, MAP decreased significantly during incision of skin and dura (*P* < 0.05) and intraoperative opoid consumption was remarkably reduced (*P* < 0.01). Postoperative remedial analgesia was earlier in the NSB group (*P* < 0.001). No patients reported any adverse events.

**Conclusion:**

Among patients undergoing craniotomy for vestibular schwannoma via a suboccipital retrosigmoid approach, LOGAB may be a promising treatment for perioperative analgesia and has the potential to maintain intraoperative hemodynamic stability.

**Clinical trial registration number:**

Chictr.org.cn ChiCTR2000038798.

## Introduction

Postoperative pain of neurosurgery has been traditionally underestimated, and patients undergoing craniotomy often recieved inadequate analgesia [1]. A prospective study of 256 patients showed that 87% of patients experienced acute pain after craniotomy, of which 32% had mild pain, 44% had moderate pain, and 11% had severe pain [2]. Increased blood pressure caused by postoperative pain may cause adverse events such as elevated intracranial pressure and increased risk of postoperative bleeding [3]. 

**Fig. 1:**
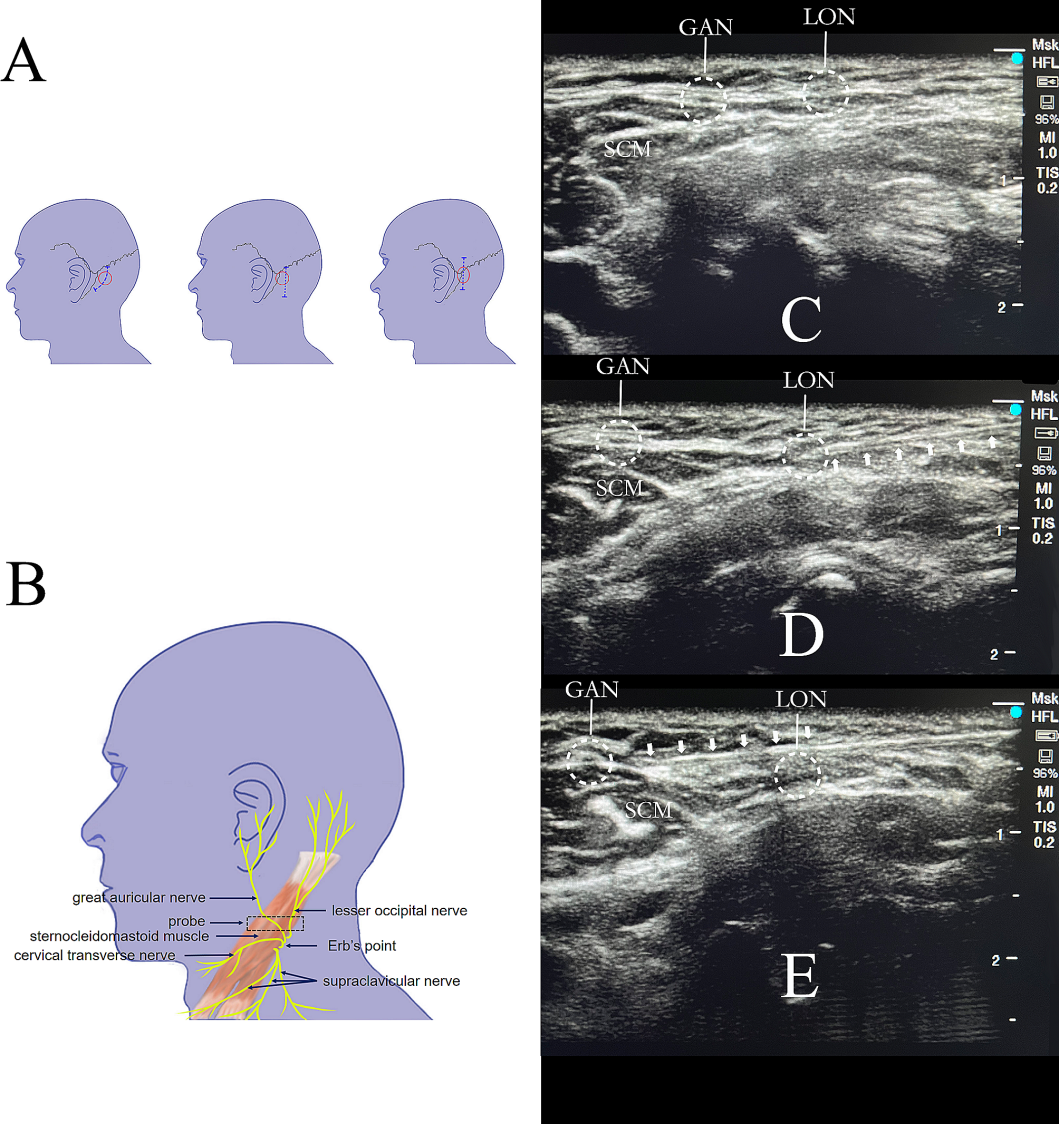
Surgical incisions, anatomy of cervical plexus and ultrasound-guided lesser occipital nerve combined with great auricular nerve block. (**A**) Three types of posterior suboccipital retrosigmoid incisions for vestibular schwannoma craniotomy. The red circle indicates the tumor site, and the blue dashed line indicates the commonly used incisions. (**B**) Branches of the cervical plexus and placement of the ultrasound probe for the lesser occipital nerve combined with great auricular nerve block. (**C**) Ultrasound images of lesser occipital nerve and greater auricular nerve. (**D**) Ultrasound-guided lesser occipital nerve block. (**E**) Ultrasound-guided greater auricular nerve block. GAN: greater auricular nerve; LON: lesser occipital nerve; SCM: Sternocleidomastoid muscle. The white arrows indicate the shaft of block needle

Previous studies showed that 41–84% of neurosurgical patients have experienced moderate to severe pain that requires potent analgesics [1, 4, 5]. Multimodel strategies have been explored to prevent the occurrence of postoperative pain following craniotomy, including opioids, non-steroidal anti-inflammatory drugs, anticonvulsants, scalp block, and local infiltration, etc. [6–9]. However, excessive opioids may interfere with the postoperative evaluation of neurological function, and induce respiratory depression, nausea and vomiting [10]. A suboccipital retrosigmoid incision is a minimally invasive approach for the treatment of tumors in cerebellopontine angle (CPA) zone. The incision is located in the subtentorial area [11, 12]. Patients undergoing infratentorial craniotomy reported more severe pain than supratentorial craniotomy on the first postoperative day, both at rest and during movement, and subsequently required more opioids or analgesics [1]. Scalp block alleviates supratentorial pain except subtentorial area that results in patients’ dissatisfaction [13]. The incision of suboccipital retrosigmoid extends along the ear and mastoid longitudinally or arcuately (Fig. 1A), and mainly involves subtentorial zone [14]. Anatomically, lesser occipital nerve block combined with greater auricular nerve block can effectively interrupt the pain signal of subtentorial area (Fig. 1B) [15–17]. A recent study has adopted intermediate cervical plexus block for posterior fossa craniotomy [18]. However, phrenic nerve blockage following intermediate cervical plexus block was as high as 58% which significantly deteriorated patients’ lung function [19, 20]. Therefore, a subcutaneous approach of lesser occipital block and greater auricular nerve block may reduce the incidence of phrenic nerve paralysis. Patients with minimally invasive resection of vestibular schwannoma were selected as the study subjects because 80% of the tumors in the cerebellopontine angle were vestibular schwannomas [21]. 

In this prospective, double-blind, randomized trial, patients with vestibular schwannoma craniotomy via the retrosigmoid suboccipital approach were treated preoperatively with ultrasound-guided lesser occipital nerve block combined with greater auricular nerve block (LOGAB). We hypothesized that LOGAB can reduce intraoperative opioid requirement and posteroperative NRS scores. The aim of this study was to evaluate the analgesic effect of ultrasound-guided LOGAB on the postoperative pain after vestibular schwannoma resection via a suboccipital retrosigmoid approach.

## Methods

### Study design

This was an investigator-initiated, prospective, single-center, double-blind, randomized, placebo-controlled trial. The trial protocol was approved by the Ethics Committee of Tongji Medical College, Huazhong University of Science and Technology (TJ2020S143; date of approval: 20/09/2020) and the written informed consent was obtained from all subjects participating in this trial. The trial was registered prior to patient enrollment at http://www.chictr.org.cn (number: ChiCTR2000038798; date of registration: 02/10/2020). An independent data and safety monitoring committee oversaw the study conduct and reviewed blinded safety data. This study was conducted between October 12, 2020 and September 20, 2022 at Tongji Hospital affiliated to Tongji Medical College of Huazhong University of Science and Technology according to the Helsinki manifesto. This manuscript adheres to the applicable CONSORT guidelines.

### Study participants

Patients were eligible for participation if they were aged between 18 and 64 years, and scheduled to craniotomy. American Society of Anesthesiologists (ASA) Physical Status of class I, II, or III was included. The exclusion criteria included emergency operation, coagulation disorders, neuropathic disorders, allergy to local anesthetics, any chronic pain conditions, chronic opioid usage, alcohol or drug addiction, psychiatric disorders, severe liver and kidney dysfunction, history of craniotomy, pregnant and lactating women, inability to understand pain scoring, preexisting nausea and vomiting, abnormal surgical scalp sensation, and participating with other studies within 30 days.

### Randomization and blinding

Enrolled patients were randomly assigned to either the LOGAB group or the control group in a 1:1 ratio. Randomization numbers were computer-generated and concealed in sealed, opaque envelopes. One researcher opened the envelope after patient enrollment, and prepared the drugs (0.5% ropivacaine or normal saline), and delivered them to the anesthesiologist for block who was blinded. Physicians responsible for intraoperative management, data collection and postoperative follow-up was blinded to group assignments.

### Interventions

The ultrasound-guided unilateral lesser occipital nerve combined with great auricular nerve block was performed by experienced J Liu. After induction, superficial cervical plexus (C4 level) was scanned transversely by ultrasonography (Sonosite Edge II, Fujifilm, USA) with a 6 to 13 MHz ultrasound probe (linear array HFL38xi) at the midpoint of the posterior margin of sternocleidomastoid muscle (Fig. 1B). Then, the probe slid toward the cephalic side 1 ∼ 2 cm to acquire clear image of both lesser occipital nerve and greater auricular nerve (Fig. 1C). Under aseptic conditions, a 22-gauge 50-mm needle (B. Braun Stimuplex D, Meisungen, Germany) was advanced twice into the subcutaneous space using “in-plane” method, outside the investing fascia (Fig. 1D and E). Water separation was utilized throughout the insertion to avoid nerve damage. Correct insertion of needle was verified by enhancement of nerve bundles after injection. A repeat negative aspiration test was performed during injection.

5 ml of 0.5% ropivacaine in the LOGAB group and an equal volume of 0.9% normal saline in the NSB group was injected through the posterolateral approach. The blocking procedure was completed within 10 min in both groups. Patients in both groups received local infiltration with 0.5% ropivacaine at head fixation sites before surgery.

### Intraoperative and postoperative care

No premedication was given before surgery. Patients were fitted by standard monitoring including heart rate (HR), continuous pulse oximetry, invasive arterial blood pressure (IABP) via arteria dorsalis pedis, continuous capnography, body temperature and bispectral index (BIS). General anesthesia was induced with intravenous propofol (2 mg ∙ kg^–1^) and sufentanil (0.4 μg ∙ kg^–1^). Rocuronium (0.9 mg ∙ kg^–1^) was intravenously administrated to facilitate endotracheal intubation. Dexamethasone (10 mg) and flurbiprofen axetil (50 mg) were intravenously administrated immediately after induction. Mechanical ventilation was initiated with a tidal volume of 6 ∼ 8 ml ∙ kg^–1^ in air-oxygen mixed gas (60% oxygen) and a respiratory rate of 12 ∼ 15 per minute to maintain EtCO_2_ between 35 and 40 mmHg. General anesthesia was maintained with sevoflurane inhalation at 0.5 minimal alveolar concentration (MAC) and a continuously intravenous infusion of remifentanil (0.1 ∼ 0.5 μg ∙ kg^–1^ ∙ min^–1^) to maintain BIS values between 40 and 60. The remifentanil rate was adjusted when MAP and/or HR fluctuated beyond 20% of baseline levels. Sufentanil (0.1 ∼ 0.2 μg ∙ kg^–1^) was remedially administered to relieve potent surgical stimulations. Clinical data including operation and anesthesia time, urine volume, blood loss, incision length were recorded. Tropisetron (2 mg) was intravenously administered before extubation. The intensity of postoperative pain was assessed using numeric rating scale (NRS) score after surgery. If NRS score > 4, a bolus of anal diclofenac (12.5 mg) was used as the remedial medication to avoid postoperative opioid interference with neurological evaluation. Time of diclofenac administration and the total dosage were recorded.

### Outcomes

Primary outcome was the NRS scores at rest and motion within 48 h after surgery. The NRS score was assessed using a measuring ruler of 10 cm length. All patients received NRS scoring training during the preoperative consultation: 0 for completely painless and 10 for unbearable worst pain. Postoperative pain was assessed at extubation, and at six different time points (2, 4, 6, 12, 24, 48 h) after surgery.

Secondary outcomes included MAP and HR data from several intraoperative time points: 1 min before and after anesthesia induction; 1 min before and after skin incision; 3 min, 5 min, 10 min after skin incision; 1 min before and after dural incision; 3 min, 5 min, 10 min after dural incision. Besides, the intraoperative opioid consumption including the average remifentanil rate, total dosage of remedial sufentanil the time of diclofenac administration, the total dosage of drug and RSS score within 48 h after surgery were recorded as secondary outcomes either. RSS score was documented through the following grading: (1) anxiety; (2) awake and quiet; (3) lethargy, but able to follow instructions; (4) falling asleep, but arousable; (5) falling asleep, slow response to verbal stimuli; (6) deep sleep, not arousable. Adverse events including hematoma, infection, dyspnea, and local anesthetics toxicity, postoperative nausea and vomiting were conducted at the same time as the postoperative NRS evaluation.

### Statistical analysis

The sample size was estimated using Power Analysis and Sample Size Software V23.0 (PASS, Kaysville, Utah, USA). Previous studies have shown that within 48 h after surgery, pain scores with and without block were 2.0 ± 1.6 and 3.7 ± 2.4, respectively [22]. Therefore, using a unilateral α level of 0.05 and 80% power, we calculated that 28 patients per group would be needed. Considering 20% loss of follow-up or consent withdrawals, 62 patients were required.

Data was analyzed on an intention-to-treat basis using the Statistical Package for Social Sciences for Windows software version 23 (IBM SPSS, USA). No patients withdrew their consent before surgery. Therefore, patients who met the inclusion criteria were included in the final analysis.

The demographic and clinical characteristics of patients were described. Normally distributed continuous variables (age, weight, height, BMI, etc.) were expressed as mean with standard difference, and analyzed using the One-Way Analysis of Variance (ANOVA) or Mann-Whitney U test. The normality of data was analyzed using the Kolmogorov-Smirnov test. Categorical variables (American Society of Anesthesiologists Physical Status, diclofenac count, postoperative nausea and vomiting, etc.) were expressed as frequency (%), and analyzed using the pearson chi-square test or Fisher’s exact test. The NRS scores at rest and motion within 48 h were plotted for homogeneity of variances test. Other variables (time, volume, rate, length, drug dosage, etc.) were processed with normality test. The ANOVA was used if data was distributed normally, or nonparametric test Mann-Whitney U test was used. Then the area under the receiver operating characteristic curve (AUC) was estimated for assessing pain intensity and duration using GraphPad Prism V.8 (GraphPad Software, La Jolla, California, USA). The mean AUCs (0 ∼ 6 h, 6 ∼ 12 h, 12 ∼ 24 h, 24 ∼ 48 h) were compared using the ANOVA according to randomization. Changes in MAP, HR and its increment rate (growth rate after skin or dural incision) were estimated by AUCs and compared by ANOVA. MAP and HR difference at time point were further analyzed. A Kaplan-Meier survival curve was generated to evaluate the difference in the first administration of diclofenac after surgery, and hazard ratio (HR) was used to evaluate the benefit using log-rank test. All tests were two-tailed, and *P* value < 0.05 was considered as significant.

## Results

### Patients

**Fig. 2:**
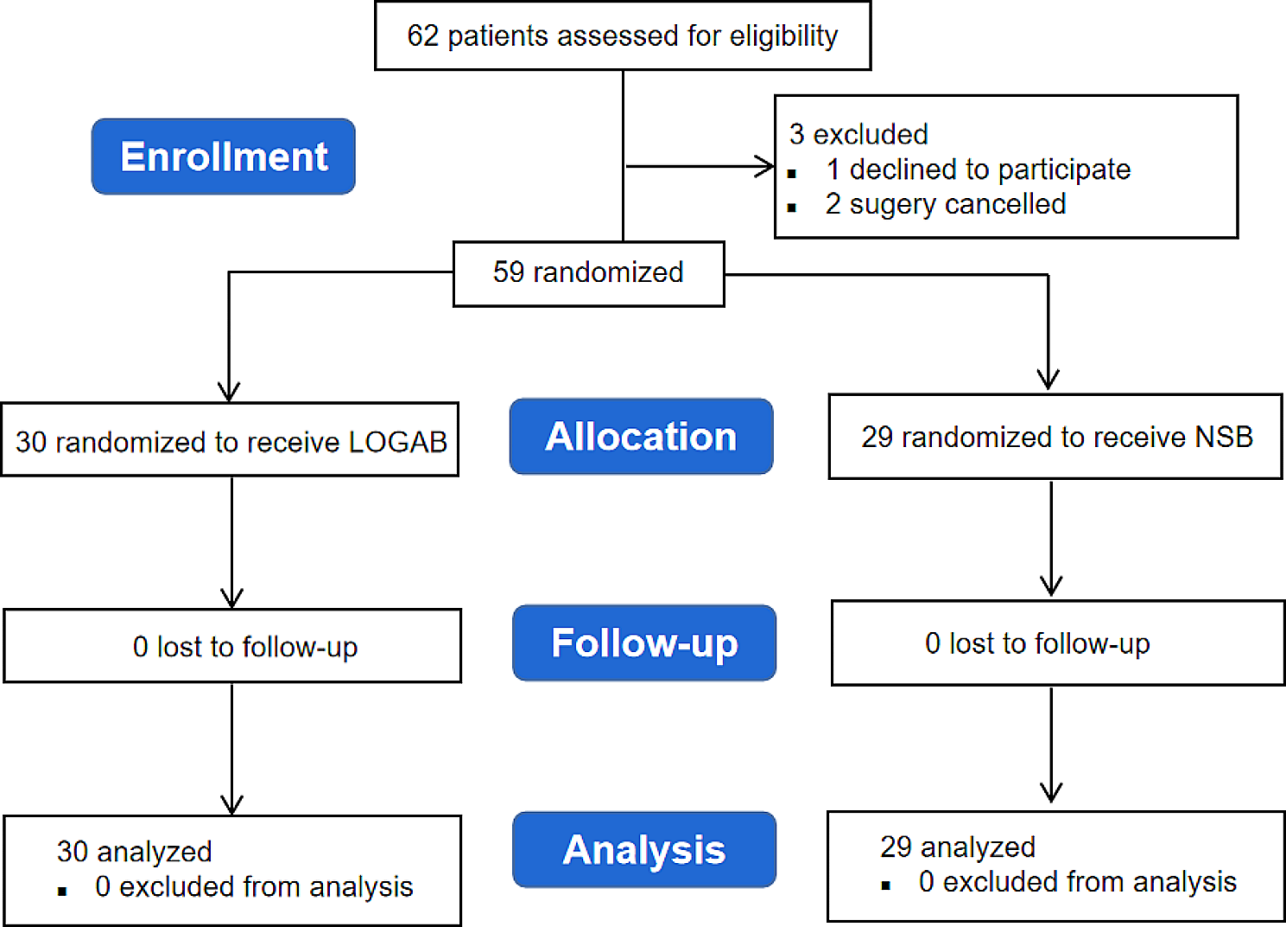
Flow of participants through the study. LOGAB: lesser occipital nerve combined with great auricular nerve block; NSB: normal saline block

A total of 62 patients were assessed for eligibility and eventually randomly assigned to either the LOGAB group (*n* = 31) or NSB group (*n* = 31). Three patients were excluded, including one patient who declined to participate in the study and two patients whose surgeries were canceled (Fig. 2). The baseline characteristics of patients including gender, age, height, weight, BMI, and ASA classification were comparable between the two groups (Table 1). No nerve block failure was reported in the two groups.

**Table 1:** Demographic and clinical characteristics

Characteristic	LOGAB Group	NSB Group	*P* value
	(*n* = 30)	(*n* = 29)	
Gender (M/F), n	10/20	11/18	0.712
Age, yr	47.4 ± 12.9	48.6 ± 11.8	0.706
Weight, kg	60.1 ± 9.8	61.7 ± 10.1	0.520
Height, cm	161.9 ± 6.9	162.8 ± 7.5	0.611
BMI, kg ⋅ m^− 2^	22.8 ± 2.8	23.2 ± 3.2	0.598
ASA, No. (%)			
I II III	4 (13) 21 (70) 5 (17)	1 (3) 25 (86) 3 (10)	0.199
Duration of surgery, min	370.6 ± 99.5	373.2 ± 99.0	0.931
Duration of anesthesia, min	427.6 ± 99.7	414.7 ± 100.4	0.670
Length of incision, cm	9.7 ± 2.7	8.0 ± 1.9	0.092
Tumor size, ml	16.9 ± 11.7	18.3 ± 7.6	0.805
Intraoperative blood loss, ml	386.1 ± 202.8	418.2 ± 263.9	0.715
Intraoperative urinary volume, ml	1104.3 ± 359.3	1246.4 ± 372.3	0.258

The data are presented as mean ± SD for continuous variables and numbers (percentages) for categorical variables

LOGAB: lesser occipital nerve combined with great auricular nerve block; NSB: normal saline block. BMI: body mass index; ASA: American Society of Anesthesiologists; SD: standard deviation

### Primary outcome

**Fig. 3:**
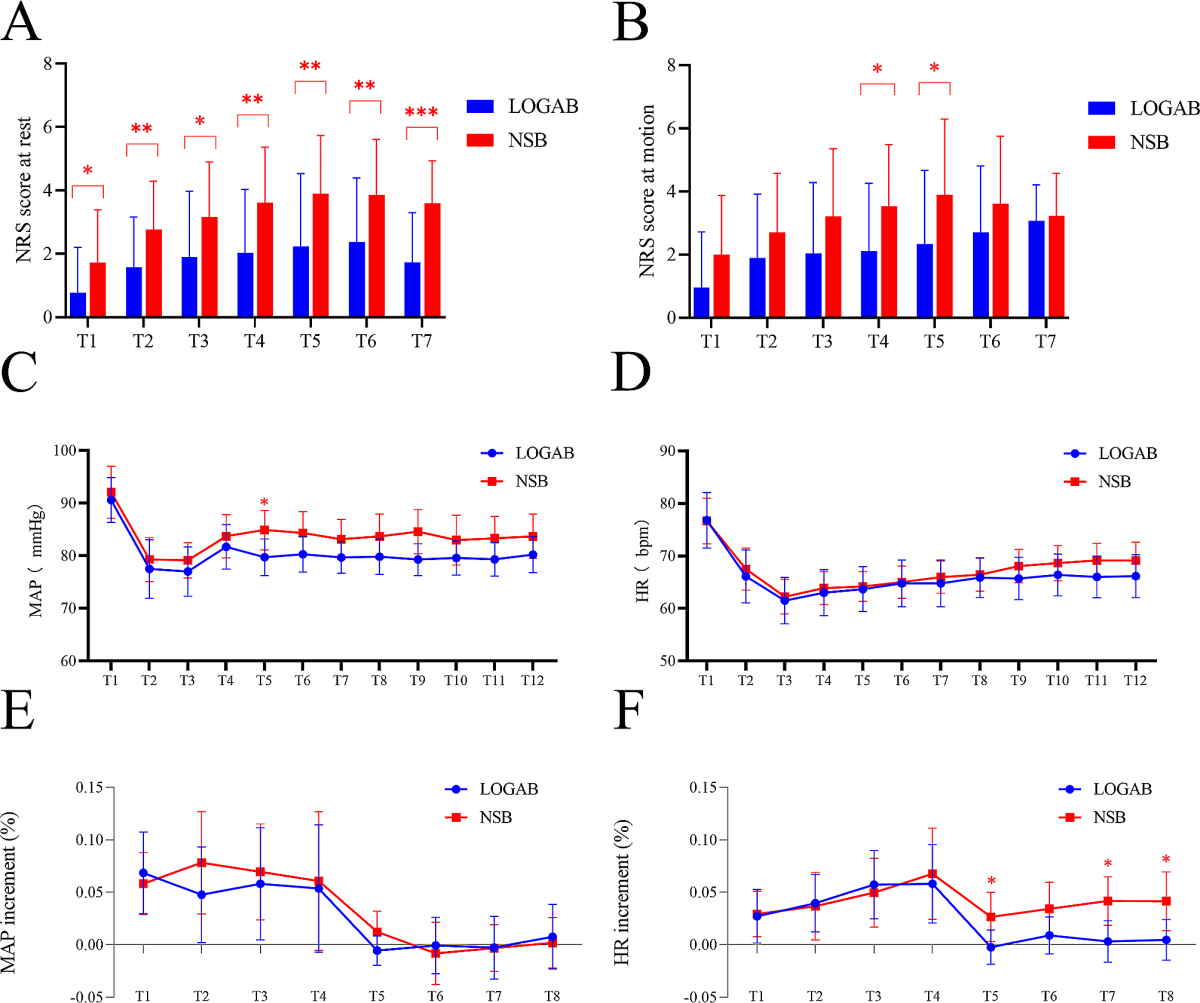
Box plots of NRS scores within 48 h after surgery, intraoperative MAP and HR and their increment rates during skin and dural incisions. (**A**) NRS scores at rest. (**B**) NRS scores at motion. NRS, Numeric Pain Rating Scales; LOGAB, ultrasound-guided unilateral lesser occipital nerve combined with great auricular nerve block; NSB, normal saline block. T1, extubation; T2, 2 h after surgery; T3, 4 h after surgery; T4, 6 h after surgery; T5, 12 h after surgery; T6, 24 h after surgery; T7, 48 h after surgery. *P < 0.05, **P < 0.01, ***P < 0.001. (**C**) MAP during skin and dural Incisions. (**D**) HR during skin and dural Incisions. MAP, mean arterial pressure; HR, heart rate; LOGAB, ultrasound-guided unilateral lesser occipital nerve combined with great auricular nerve block; NSB, normal saline block. T1, 1 min before induction; T2, 1 min after induction; T3, 1 min before skin incision; T4, 1 min after skin incision; T5, 3 min after skin incision; T6, 5 min after skin incision; T7, 10 min after skin incision; T8, 1 min before dural incision; T9, 1 min after dural incision; T10, 3 min after dural incision; T11, 5 min after dural incision; T12, 10 min after dural incision. *P < 0.05. (**E**) MAP increment (%) during skin and dural Incisions. (**F**) HR increment (%) during skin and dural Incisions. T1, 1 min after skin incision; T2, 3 min after skin incision; T3, 5 min after skin incision; T4, 10 min after skin incision; T5, 1 min after dural incision; T6, 3 min after dural incision; T7, 5 min after dural incision; T8, 10 min after dural incision. *P < 0.05

The overall difference in the NRS scores after surgery across the LOGAB group vs. NSB group was 1.8 ± 0.5 vs. 3.2 ± 0.8 at rest and 2.2 ± 0.7 vs. 3.2 ± 0.6 at motion (*P* = 0.002, *P* = 0.013), respectively. Specifically, at each time point within 48 h after surgery, the NRS scores in LOGAB group at rest were lower than those in NSB group (*P* < 0.05) (Table 2; Fig. 3A). However, only at 6 and 12 h, LOGAB group had the lower NRS scores than NSB group at motion (2.1 ± 2.2 vs. 3.5 ± 2.0 and 2.3 ± 2.3 vs. 3.9 ± 2.4, respectively, both *P* < 0.05) (Table 2; Fig. 3B). We also compared the differences in NRS scores between rest and motion in each group, and the results showed no significant differences.

**Table 2:** Primary outcome analyses

Variable	Time after surgery	LOGAB Group	NSB Group	*P* value
	(hours)	(*n* = 30)	(*n* = 29)	
Overall NRS score at rest		1.8 ± 0.5	3.2 ± 0.8	0.002
NRS score at rest	0	0.8 ± 1.4	1.7 ± 1.7	0.021
	2	1.6 ± 1.6	2.8 ± 1.5	0.005
	4	1.9 ± 2.1	3.2 ± 1.7	0.013
	6	2.0 ± 2.0	3.6 ± 1.7	0.002
	12	2.2 ± 2.3	3.9 ± 1.8	0.003
	24	2.4 ± 2.0	3.9 ± 1.7	0.004
	48	1.7 ± 1.6	3.6 ± 1.4	< 0.0001
Overall NRS score at motion		2.2 ± 0.7	3.2 ± 0.6	0.013
NRS score at motion	0	1.0 ± 1.8	2.0 ± 1.9	0.072
	2	1.9 ± 2.0	2.7 ± 1.9	0.187
	4	2.0 ± 2.2	3.2 ± 2.1	0.083
	6	2.1 ± 2.2	3.5 ± 2.0	0.028
	12	2.3 ± 2.3	3.9 ± 2.4	0.033
	24	2.7 ± 2.1	3.6 ± 2.1	0.167
	48	3.1 ± 1.1	3.2 ± 1.4	0.694
Mean AUCs at rest, units × hours	0 ∼ 48	99.3 ± 85.7	175.7 ± 73.6	< 0.001
AUCs at rest, units × hours	0 ∼ 6	9.7 ± 9.5	17.2 ± 9.0	0.003
	6 ∼ 12	12.8 ± 12.7	22.6 ± 10.3	0.002
	12 ∼ 24	27.6 ± 25.4	46.6 ± 21.3	0.003
	24 ∼ 48	49.2 ± 41.3^*^	89.4 ± 36.2	< 0.001
Mean AUCs at motion, units × hours	0 ∼ 48	123.6 ± 77.1	157.5 ± 89.7	0.176
AUCs at motion, units × hours	0 ∼ 6	10.7 ± 11.2	16.2 ± 11.0	0.105
	6 ∼ 12	13.3 ± 13.1	22.3 ± 12.7	0.026
	12 ∼ 24	30.2 ± 25.7	43.7 ± 26.3	0.096
	24 ∼ 48	69.3 ± 32.3^*^	82 ± 40.4	0.519

The data are presented as mean ± SD. LOGAB, lesser occipital nerve combined with great auricular nerve block; NSB: normal saline block; NRS: Numeric Rating Scales; AUC: area under the receiver operating characteristic curve; SD: standard deviation. ^*^P < 0.05

We further proceeded AUCs of pain score for evaluating pain duration and intensity (Table 2). There was a statistically significant difference in the mean AUC_0 ∼ 48 h_ at rest between two groups, which were 99.3 ± 85.7 vs. 175.7 ± 73.6 in the LOGAB and NSB group, respectively (*P* < 0.001). Meanwhile, the mean AUC_0 ∼ 48 h_ at motion were comparable between the LOGAB and NSB groups (123.6 ± 77.1 vs. 157.5 ± 89.7, *P* > 0.05). Subgroup analyses of the AUCs of NRS scores at each 6 h interval (Table 2). There were significant differences in the AUC_0 ∼ 6 h_, AUC_6 ∼ 12 h_, AUC_12 ∼ 24 h_ and AUC_24 ∼ 48 h_ (*P* < 0.01, *P* < 0.01, *P* < 0.01, *P* < 0.001, respectively) at rest between the LOGAB and NSB groups. No significant difference was observed in the AUC_0 ∼ 6 h_, AUC_12 ∼ 24 h_ or AUC_24 ∼ 48 h_ at motion (both *P* > 0.05), while the AUC_6 ∼ 12 h_ in the LOGAB group at motion was lower than that in the NSB group (*P* < 0.05). In addition, we also confirmed that the AUCs between rest and motion state in each group were comparable except the AUC_24 ∼ 48 h_ in the LOGAB group (*P* < 0.05).

### Secondary outcomes

As shown in Fig. 3C, during the periods of skin and dural incisions, the overall MAP was significantly lower in the LOGAB group than that in the NSB group (*P* = 0.011), especially at 3 min after skin incision (*P* = 0.044) and 1 min after dural incision (*P* = 0.039). There were no significant differences in HR at any time points throughout surgery between the two groups (*P* > 0.05) (Fig. 3D). Then, the increment rate of MAP and HR at each time point before and after skin or dural incision were further analyzed. The results showed that there was no significant difference in the increment rate of MAP before and after skin or dural incision between the two group (Fig. 3E). HR increment rates in the LOGAB group at 1 min, 5 min, and 10 min after dural incision were lower than those in the NSB group, respectively (*P* = 0.042, *P* = 0.012, and *P* = 0.030, respectively) (Fig. 3F). Specifically, within the 10 min after skin and dural incision, 16 of 30 patients (53.3%) in the LOGAB group and 10 of 29 patients (34.5%) in the NSB group had an increment of MAP > 20%, respectively. And 9 of 30 patients (30%) in the LOGAB group and 10 of 29 patients (34.5%) in the NSB group had an increment of HR > 20%, respectively. (both *P* > 0.05, Table 3).

**Table 3:** Secondary outcome analyses

Variable	LOGAB Group	NSB Group	*P* value
	(*n* = 30)	(*n* = 29)	
MAP increment > 20%, No. (means, interquartile range)	16 (30%, 21–39%)	10 (36%, 22–47%)	0.293
HR increment > 20%, No. (means, interquartile range)	9 (26%, 22–30%)	10 (29%, 24–32%)	0.355
Infusion rate of remifentanil, 10^− 3^ μg. kg^− 1^. min^− 1^	99.5 ± 20.9	137.6 ± 73.3	0.008
Sufentanil remedy, μg	7.9 ± 2.7	26.1 ± 11.1	0.001
Number of patients who needed diclofenac, No. (%)	9 (30%)	19 (66%)	0.006
Dosage of diclofenac, mg	18.1 ± 6.6	18.4 ± 7.6	0.903
Postoperative RSS score	2.6 ± 0.4	2.6 ± 0.5	0.871
Postoperative nausea, No. (%)	9 (30%)	14 (48%)	0.150
Postoperative vomiting, No. (%)	10 (33%)	8 (28%)	0.632

The data are presented as mean ± SD for continuous variables and numbers (percentages) for categorical variables. LOGAB, lesser occipital nerve combined with great auricular nerve block; NSB: normal saline block; MAP: mean arterial pressure; HR: heart rate; RSS: ramsay sedation score; SD: standard deviation

The average remifentanil rate in the LOGAB group was significantly lower than that in the NSB group (*P* = 0.008). Meanwhile, intraoperatively remedial sufentanil was also remarkably lower in the LOGAB group than that in the NSB group (*P* = 0.001) (Table 3).

Within 48 h after surgery, 9 of 30 patients (30%) in the LOGAB group and 19 of 29 patients (65.5%) in the NSB group required diclofenac, respectively (*P* < 0.01), while the mean diclofenac dose between two groups were comparable (*P* > 0.05) (Table 3). The Kaplan-Meier curves suggested that patients in the NSB group needs the first administration of diclofenac earlier than patients in the LOGAB group after surgery (*P* < 0.001) .

There was no significant difference in the duration of surgery, duration of anesthesia, length of incision, tumor size, intraoperative blood loss, or intraoperative urine volume between the two groups (*P* > 0.05).

The postoperative RSS scores were comparable between LOGAB and NSB groups (2.6 ± 0.4 vs. 2.6 ± 0.5, *P* > 0.05). Incidence of postoperative nausea and vomiting within 48 h after surgery were similar among two groups (*P* > 0.05). No adverse events such as injection point hematoma, infection, dyspnea, or systemic toxicity of local anesthetics were reported in all the patients.

## Discussion

In this study, we conducted a prospective, double-blind randomized controlled trial to assess a strategy of ultrasound-guided LOGAB for analgesia in vestibular schwannoma craniotomy via a suboccipital retrosigmoid approach. The use of LOGAB significantly reduced perioperative pain and opioid consumption. Hemodynamics fluctuation can lead to perioperative complications of craniotomy [23]. However, according to our study, we found that LOGAB may have potential benefits for maintaining hemodynamic stability and achieving a reduction in analgesic requirements within 48 h after surgery.

Superficial cervical plexus block has been developed for decades, and generally used for analgesia in neck, shoulder and clavicle [24–26], as well as for ear surgery [27]. Based on the latest neuroanatomical classification cervical plexus can be subdivided into three anatomical categories, which include the superficial cervical plexus (Erb’s point, between the skin and the superficial layer of investing fascia) [28], the middle cervical plexus (between the deep layer of the investing fascia and the prevertebral fascia), and the deep cervical plexus (between the prevertebral fascia and scalene muscle fascia). Incidences of hemi-phrenic paralysis in three types of cervical plexus block are 13%, 93%, and 93%, respectively [29], which may lead to respiratory complications related to nerve block. Based on the anatomic innervation of postauricular region adopted previously [15, 30], we proposed that visualized blockage using ultrasound of the lesser occipital nerve and greater auricular nerve can provide analgesia covering the retroauricular region. To our knowledge, this is the first study using LOGAB for retroauricular craniotomy. Our results have proved that LOGAB could provide sufficient analgesic effects. In addition, the other branches of cervical plexus (transverse cervical nerve and supraclavicular nerve) may be avoided blocking by moving needle up 1 to 2 cm away from the Erb’s point.

Our study has confirmed that ultrasound-guided LOGAB reduced the consumption of opioids including remifentanil and sufentanil, which might reduce the incidence of postoperative hyperalgesia [31, 32]. Within 48 h after surgery, patients receiving LOGAB had significantly lower NRS scores at rest. However, a prospective randomized controlled study of bilateral scalp block for supratentorial craniotomy by Rigamonti et al. showed that nerve block could only reduce the Visual Analogue Scale (VAS) scores within 4 h after surgery, while no analgesic effect was observed at 8 ∼ 48 h [33]. They believe that local anesthetic can be rapidly absorbed since scalp is rich in vessels. In our study, LOGAB was performed subcutaneously, resulting an analgesia up to 48 h. Pain at motion was significantly reduced in LOGAB group, especially at 6th and 12th hours after surgery. One possible explanation is the slow onset of motor blocks caused by local anesthetics [18]. 

Different from a previous study by M Zeng et al., [34] we used local anesthetic infiltration at skull pin fixation sites to prevent potent stimulation instead of additional sufentanil. Our results showed that the overall NRS scores at rest and motion within 48 h in the LOGAB group were reduced by 1.4 and 1, respectively, while M Zeng’s study presented the decreases of 0.5 and 0.6, indicating the importance of local anesthetic injection. The AUCs of pain scores can be used for the interpretation of pain intensity and duration [35]. In our study, resting AUCs in LOGAB group was significantly decreased which indicates a continuous analgesic effect within 48 h after surgery. Even though there was no difference at motion, a further subgroup analysis showed that there was a lower AUC_6 ∼ 12 h_ in the LOGAB group at motion. A plausible explanation is that intravenous analgesics may have affected the assessment of pain early after surgery.

In this study, rescue analgesia after surgery was applied with diclofenac instead of postoperative patient-controlled analgesia (PCA) to avoid the impact on neurological function assessment and reduce the side effects of dizziness, nausea and vomiting. Fewer patients required rescue analgesia in the LOGAB group, but the mean dosage of diclofenac were comparable. In addition, first administration of diclofenac in NSB group was much earlier. These suggest that a longer analgesic duration of LOGAB.

The beneficial effects of scalp block was abundantly described on hemodynamic stability during skull pin fixation, noxious skin and dural incision in supratentorial craniotomy [9]. In our study, local infiltration was performed at skull pin fixation sites before surgery. We found that LOGAB reduced the remifentanil infusion rate to 0.056 μg/kg/min, while Michele et al. reported similar findings that scalp block reduced the remifentanil infusion rate to 0.050 μg/kg/min in supratentorial craniotomy. Remifentanil rate is much lower than 0.3 μg/kg/min, which can significantly reduce the incidence of burst pain caused by discontinuing remifentanil administration [36]. In our study, LOGAB exhibited an opioid sparing effect. The analgesic effect of LOGAB for subtentorial surgery was similar as scalp block for supratentorial craniotomy [37]. 

In our study, intraoperative MAP was relatively lower in patients receiving LOGAB, especially at 3 min after skin incision and 1 min after dural incision. Muscle dissection and dural traction may be potent stimuli to induce MAP elevation [38]. Patients without block experienced hyperalgesia after skin incision [32], resulting in elevated MAP. However, the MAP increment rate did not differ between groups. Besides, we observed a lower HR increment rate in LOGAB group at 1, 5, 10 min after dural incision, suggesting that LOGAB inhibited MAP elevation and HR variation. The skin and subcutaneous tissue in CPA region are anatomically innervated by lesser occipital nerve and great auricular nerve. Studies have shown that the trigeminal nerve and the C1-C3 cervical nerve give off communicating branches at the dura and jointly innervate the dura of the posterior cranial fossa [39]. The above evidence suggests that LOGAB may block the nerve branches innervating the surgical incision of vestibular schwannoma and the corresponding dura. Further anatomical studies should be proceeded.

However, our study still has limitations. Firstly, the study sample size was small, so the difference in NRS at motion between the two groups was not significant. Secondly, CPA tumors are inherently characterized by vertigo and nausea, affecting the evaluation of opioid side effects. Thirdly, in this study, the sample size was calculated without distinguishing between resting or exercise pain scores, so there may be selection bias. Fourthly, the visibility of lesser occipital nerve and greater auricular nerve are usually difficult to identify on ultrasound, and hydro-dissection technique would be helpful.

## Conclusion

Our study suggests that LOGAB may be a promising treatment for perioperative analgesia and has the potential to maintain intraoperative hemodynamic stability for vestibular schwannoma craniotomy via a suboccipital retrosigmoid approach. Further studies with large samples are demanded to explore the effect of LOGAB on perioperative pain.

## Data Availability

No datasets were generated or analysed during the current study.

## Abbreviations

AUC: Area under curve
CPA: Cerebellopontine angle
HR: Heart rate
LOGAB: Lesser occipital nerve combined with great auricular nerve block
MAP: Mean arterial pressure
NRS: Numeric Rating Scales
NSB: Normal saline block
PCA: Patient-Controlled Analgesia
RSS: Ramsay Sedation Score
VAS: Visual Analogue Scale
